# The clinical features, treatment and prognosis of neutropenic fever and Coronavirus disease 2019 results of the multicentre teos study

**DOI:** 10.1038/s41598-024-55886-w

**Published:** 2024-03-03

**Authors:** Dilşah Başkol Elik, Şafak Kaya, Sevil Alkan, Tuna Demirdal, Alper Sener, Selçuk Kaya, Özlem Güzel Tunçcan, Bircan Kayaaslan, Rahmet Güner, Fatma Eser, Hasip Kahraman, Serhat Birengel, Elif Mukime Sarıcaoğlu, Esma Eroğlu, Fatma Çölkesen, Erman Öztürk, Hande Berk Cam, Çiğdem Mermutluoğlu, Şafak Özer Balin, Gülden Sincan, Nilgün Altın, Uluhan Sili, Bedia Mutay Suntur, Tuğba Arslan Gülen, Burak Deveci, Rabin Saba, Şaban İncecik, Gülden Eser Karlıdağ, Elif Hakko, Damla Akdağ, Hüseyin Aytaç Erdem, Hilal Sipahi, Candan Çicek, Mehmet Sezai Taşbakan, Meltem Taşbakan, Hüsnü Pullukçu, Tansu Yamazhan, Bilgin Arda, Sercan Ulusoy, Oguz Resat Sipahi

**Affiliations:** 1 Department of Infectious Disease and Clinical Microbiology, Faculty of Medicine, Ege University, Bornova, Izmir, Turkey; 2 Department of Infectious Diseases, Gazi Yaşargil Training and Research Hospital, University of Health Sciences, Diyarbakir, Turkey; 3 Department of Infectious Disease, Faculty of Medicine, Canakkale Onsekiz Mart University, Canakkale, Turkey; 4 Department of Infectious Disease and Clinical Microbiology, Faculty of Medicine, Izmir Katip Celebi University, Izmir, Turkey; 5 Department of Infectious Disease and Clinical Microbiology, Faculty of Medicine, Karadeniz Technical University, Trabzon, Turkey; 6 Department of Infectious Disease and Clinical Microbiology, Faculty of Medicine, Gazi University, Ankara, Turkey; 7 Department of Infectious Disease and Clinical Microbiology, Ankara City Hospital, Ankara Yıldırım Beyazit University, Ankara, Turkey; 8 Department of Infectious Disease and Clinical Microbiology, Faculty of Medicine, Eskisehir Osmangazi University, Eskisehir, Turkey; 9 Department of Infectious Disease and Clinical Microbiology, Faculty of Medicine, Ankara University, Ankara, Turkey; 10 Department of Infectious Disease and Clinical Microbiology, Konya Meram State Hospital, Konya, Turkey; 11 Department of Hematology, Faculty of Medicine, Istanbul Medeniyet University, Istanbul, Turkey; 12 Department of Infectious Disease and Clinical Microbiology, Antalya Training and Research Hospital, University of Health Sciences, Antalya, Turkey; 13 Department of Infectious Disease and Clinical Microbiology, Faculty of Medicine, Dicle University, Diyarbakir, Turkey; 14 Department of Infectious Disease and Clinical Microbiology, Faculty of Medicine, Firat University, Elazig, Turkey; 15 Department of Hematology, Faculty of Medicine, Atatürk University, Erzurum, Turkey; 16 Department of Infectious Disease and Clinical Microbiology, Dışkapı Yıldırım Beyazıt Training and Research Hospital, University of Health Sciences, Ankara, Turkey; 17 Department of Infectious Disease and Clinical Microbiology, Faculty of Medicine, Marmara University, Istanbul, Turkey; 18 Infectious Diseases, Adana City Training and Research Hospital, Adana, Turkey; 19 Department of Hematology and Stem Cell Transplant Unit, Medstar Antalya Hospital, Antalya, Turkey; 20 Department of Infectious Disease and Clinical Microbiology, Faculty of Medicine, Van Yüzüncü Yıl University, Van, Turkey; 21 Department of Infectious Disease and Clinical Microbiology, Elazığ Fethi Sekin City Hospital, University of Health Sciences, Elazig, Turkey; 22 Department of Infectious Disease and Clinical Microbiology, Anadolu Medical Center, Istanbul, Turkey; 23 Department of Infectious Diseases and Clinical Microbiology, Basaksehir Cam Sakura City Hospital, Istanbul, Turkey; 24 Bornova Directorate of Health, Bornova, Izmir, Turkey; 25 Department of Medical Microbiology, Faculty of Medicine, Ege University, Bornova, Izmir, Turkey; 26 Department of Chest Diseases, Faculty of Medicine, Ege University, Bornova, Izmir, Turkey; 27 Department of Infectious Diseases, Bahrain Oncology Center, King Hamad University Hospital, Muharraq, Bahrain; 28 Infectious Disease and Clinical Microbiology, Turgutlu State Hospital, Manisa, Turkey

**Keywords:** SARS-CoV-2, Antiviral agents, Clinical microbiology, Cancer, Haematological cancer

## Abstract

This multicentre (22 centres in Turkey) retrospective cohort study aimed to assess the clinical outcomes of patients with neutropenic fever and SARS-CoV-2 positivity. Study period was 15 March 2020–15 August 2021. A total of 170 cases (58 female, aged 59 ± 15.5 years) that fulfilled the inclusion criteria were included in the study. One-month mortality rate (OMM) was 44.8%. The logistic regression analysis showed the following significant variables for the mentioned dependent variables: (i) achieving PCR negativity: receiving a maximum of 5 days of favipiravir (*p* = 0.005, OR 5.166, 95% CI 1.639–16.280); (ii) need for ICU: receiving glycopeptide therapy at any time during the COVID-19/FEN episode (*p* = 0.001, OR 6.566, 95% CI 2.137–20.172), the need for mechanical ventilation (*p* < 0.001, OR 62.042, 95% CI 9.528–404.011); (iii) need for mechanical ventilation: failure to recover from neutropenia (*p* < 0.001, OR 17.869, 95% CI 3.592–88.907), receiving tocilizumab therapy (*p* = 0.028, OR 32.227, 95% CI 1.469–707.053), septic shock (*p* = 0.001, OR 15.4 96% CI 3.164–75.897), and the need for ICU (*p* < 0.001, OR 91.818, 95% CI 15.360–548.873), (iv) OMM: [mechanical ventilation (*p* = 0.001, OR 19.041, 95% CI 3.229–112.286) and septic shock (*p* = 0.010, OR 5.589,95% CI 1.509–20.700)]. Although it includes a relatively limited number of patients, our findings suggest that COVID-19 and FEN are associated with significant mortality and morbidity.

## Introduction

The World Health Organization (WHO) declared Coronavirus disease 2019 (COVID-19) which is caused by SARS-CoV-2 (severe acute respiratory syndrome coronavirus 2) as a pandemic on March 11, 2020^1–3^. COVID-19 keeps being a serious worldwide problem with recurring outbreaks and fluctuations, with over 651 million reported cases and more than 6.6 million reported deaths as of December 23, 2022^2^. COVID-19 does not have a 100% effective treatment, yet. Although numerous vaccines have been approved and ongoing vaccination studies are underway, COVID-19 continues to pose a significant threat to humanity. Individuals with malignancies are particularly vulnerable to the virus.

Despite developments in the chemotherapy and antibiotics, febrile neutropenia or neutropenic fever (FEN) still causes significant mortality and morbidity^4^. Cancer or FEN patients are not out of global COVID-19 risk. Theoretically and practically, COVID-19 may cause or complicate FEN. During the course of COVID-19, FEN may develop, or patients may be admitted to the hospital with a clinical presentation of FEN.

Data related to FEN and COVID-19 are limited. A study published in June 2020 on the follow-up of patients with cancer during the COVID-19 pandemic process reported that granulocyte colony-stimulating factor (GCSF) might reduce FEN attacks for this group of patients^5^. In another study analysing 77 SARS-CoV-2 patients who underwent stem cell transplantation, the 30-day survival rate was found to be 78%^6^. However, there have been no published large clinical studies conducted yet on the treatment and response of COVID-19 in this high-risk subgroup.

Herein, we aimed to evaluate the demographic characteristics, clinical course, treatment response, survival, and factors associated with the survival of patients with FEN + SARS-CoV-2 polymerase chain reaction (PCR) positivity, retrospectively.

## Methods

### Study group

This multicentre retrospective cohort study gathered data from 14 cities (İzmir, Çanakkale, Adana, Diyarbakır, Trabzon, Ankara, Eskişehir, Konya, İstanbul, Antalya, Van, Erzurum, Kocaeli, Elazığ) and 22 centres located in seven regions of Turkey. The study period was 15 March 2020–15 August 2021. The study was announced in the e-mail list of Infectious Diseases and Clinical Microbiology Specialty Society of Turkey (Türkiye-EKMUD) on 15 February 2021. The work plan and Excel forms for data collection were sent via email to the participating centres^7^.

Study inclusion criteria were as follows:

- Microbiologically proven COVID-19 infection (SARS-CoV-2 PCR positivity in nasopharyngeal swab sample)
- When diagnosed with COVID-19, having FEN (absolute neutrophil count < 500 cells/mm^3^ and a fever of > 38.3 °C once or two or more 38 °C fever in 12 h)
- Aged > 18 years.

FEN risk evaluation was conducted based on the criteria of the Multinational Association for Supportive Care in Cancer (MASCC), and patients with a score of ≥ 21 were classified as low-risk cases^8^.

### Case record form

A Microsoft Excel-based case record form was filled for patients who met the study inclusion criteria. This form comprised the following parameters; stay in the intensive care unit (ICU), underlying malignancy and chronic diseases, history of chemotherapy, and COVID-19 vaccination, bone marrow transplantation, laboratory (including biochemical, microbiological, virological), radiological, and clinical findings at the time of hospital admission before the diagnosis of FEN and during COVID-19 hospitalization. Antibacterial and COVID-19 treatment regimens as well as clinical response during follow-up including the need of supplemental oxygen, ICU, mechanical ventilation, day 30 and 90 survival after treatment and reinfection rates were determined.

### Study parameter definitions

SARS-CoV-2 positivity was determined by a positive result on the real-time RT-PCR (reverse transcriptase-polymerase chain reaction) assay of nasal and/or pharyngeal swab specimens. RT-PCR was the most commonly used method worldwide during the COVID-19 pandemic^9^.

Patients who did not survive at the end of COVID-19/FEN episode treatment were classified as end of treatment (EOT) mortality. All-cause mortality during a one-month period (OMM) referred to deaths that occurred due to a documented or presumed infection, or for a known or unknown reason, within 30 days after the diagnosis of the COVID-19/FEN episode. All cause mortality during 90 days period from the first day of the COVID-19/FEN episode was defined as day 90 mortality.

Viral clearance was defined as the presence of a fever response plus at least one negative SARS-CoV-2 PCR test result along with regression of clinical symptoms. Patients were also evaluated for re-infection after the COVID-19/FEN episode. Re-infection was defined as the detection of positive SARS-CoV2 PCR again in the nasopharyngeal swab sample of individuals who developed new clinical findings at least 90 days after the initial SARS-CoV2 PCR positivity, as recommended by the Centers for Disease Control and Prevention (CDC)^9^.

Types of infection episodes other than COVID-19 were categorized as a microbiologically documented infection (MDI), clinically documented infection (CDI), or fever of unknown origin (FUO). Clinically documented infection (CDI) was considered when there was a visible source of infection during physical examination, without any microbiological evidence other than COVID-19^7,10^.

We utilized the following threshold values in laboratory result risk factor analysis: C-reactive protein (CRP) (< 75 mg/L vs. ≥ 75 mg/L, < 100 mg/L vs. ≥ 100 mg/L), d-dimer (< 1000 µg/L vs. ≥ 1000 µg/L), ferritin (< 500 µg/L vs. ≥ 500 µg/L), lactate dehydrogenase (LDH) (< 250 U/L vs. ≥ 250 U/L), and lymphocyte count (< 800/microliter vs. ≥ 800/microliter)^11,12^.

### Ethical approval

This study has approved by the Turkish Ministry of Health Scientific Research Platform (2021-03-15T22_22_01). The procedures used in this study adhere to the tenets of the Declaration of Helsinki.

### Consent to participate

Informed consent was obtained from all individual participants included in the study.

### Statistical analysis

Statistical analysis was performed via SPSS 25.0 statistical analysis program. The effect of independent univariate variables on the dependent variable was demonstrated by Chi-square analysis and Student-T test. The effect of significant independent variables on the viral clearance (achieving PCR negativity), need for ICU during the COVID-19/FEN episode, need for mechanical ventilation during the COVID-19/FEN episode, and OMM was further analysed by using binary logistic regression analysis performed via the enter method. These four variables were the dependent variables and the selected variables with a *p* < 0.05 in univariate analysis (selection criteria are detailed in supplementary material) were used as covariates. A *p* value less than 0.05 were considered to be significant.

The logistic regression analysis models performed for the PCR negativity, need for ICU during the COVID-19/FEN episode, need for mechanical ventilation during the COVID-19/FEN episode, and the OMM, achieved the overall accuracy rates of 80%, 87.1%, 92.4%, and 82.4%, respectively.

## Results

### Characteristics of the study population

A total of 170 cases (58 females, aged 59.3 ± 15.5) that fulfilled the inclusion criteria were included in the study (Table 1). The majority of the cases (n = 50, 29.40%) were from the cities in the Marmara region (Istanbul, Kocaeli and Çanakkale), where the country’s population is most populous, followed by Central Anatolia region (n = 46, 27.05%), South eastern Anatolia region (n = 19, 11.17%), Aegean region (n = 18, 10.50%), Eastern Anatolia region (n = 14, 8.23%), Mediterranean region (n = 12, 7.05%), and the Black Sea region (n = 11, 6.47%) (Table 1S in supplementary material).

**Table 1:** Characteristics of the overall study cohort.

Characteristics	Total = 170 n (%)
Female	58 (34.1%)
Age	59.3 ± 15.5
ICU	59 (34.7%)
Mechanical ventilation	44 (25.8%)
Hypotension at the time of COVID-19 diagnosis	22 (12.9%)
Vasopressor agents at the time of COVID-19 diagnosis	10 (5.9%)
High risk (MASCC score < 21) cases	71 (41.7%)
Low-risk neutropenia	99 (58.3%)
Antimicrobial therapy	
BL/BLI including empirical monotherapy	61 (35.9%)
Carbapenem including empirical monotherapy	19 (11.2%)
Combination empirical therapy with glycopeptides	74 (43.5%)
Combination empirical therapy with quinolones	24 (14.1%)
Antifungal including empirical therapy	23 (13.5%)
Symptoms	
Dyspnoea at the time of COVID-19 diagnosis	44 (25.8%)
Cough	95 (55.8%)
Diarrhoea	28 (16.5%)
Chest pain	28 (16.5%)
Headache	40 (23.5%)
Asymptomatic	16 (9.4%)

*ICU* intensive care unit, *BL/BLI* beta lactam beta lactamase inhibitor, *COVID-19* Coronavirus disease 2019, *MASCC* Multinational Association of Supportive Care in Cancer.

A total of 113 (66.5%) cases were diagnosed with COVID-19 in the hospital, while 57 cases were diagnosed outside the hospital and subsequently admitted. The mean length of hospitalization was 16.1 ± 16.3 days. The average durations from the last chemotherapy treatment and recovery from neutropenia were 27.7 ± 59.2 and 5.6 ± 4.1 days, respectively. Among the overall cohort, approximately 34.1% (58/170) had solid tumours and 54.7% (93/170) had hematologic malignancies (Table 2).

**Table 2:** Underlying malignancies and coexisting diseases of the overall study cohort.

Underlying malignancies and coexisting diseases		Total = 170 n (%)
Underlying malignancies	Hematologic malignancy	93 (54.7%)
	Solid organ tumour	58 (34.1%)
	Multiple myeloma	7 (4.1%)
	Acute myeloid leukaemia	51 (30%)
	Acute lymphocytic leukaemia	11 (6.4%)
	Chronic leukaemia	8 (4.7%)
	Lymphoma	11 (6.4%)
	Myelodysplastic syndrome	6 (3.5%)
	Other haematological malignity	8 (4.7%)
	Neutrophils < 100	38 (22.3%)
	Treatment duration (days)	16.1 ± 16.3
Coexisting diseases	Any coexisting disease	105 (61.8%)
	None	65 (38.2%)
	Diabetes mellitus	35 (20.6%)
	Chronic renal failure	7 (4.1%)
	Chronic obstructive lung disease	13 (7.6%)

### Type of infection and distribution of microorganisms other than COVID-19

Approximately 20.5% (35/170) of the FEN episodes were classified as fever of unknown origin (FUO) (no other obvious clinical/microbiological finding other than COVID-19 positivity). Meanwhile, 35.8% (61/170) were identified as having additional CDI, and 57% (97/170) experienced MDI episodes, including possible and probable Aspergillosis.

Confirmed bacterial infection-related causative microorganisms were identified in 57 cases: 34 from blood, 11 from urine, and 12 from sputum/deep tracheal aspirate cultures (Table 2). The most common causative agents other than possible/probable/definite Aspergillosis (n = 27) were *E. coli* (n = 16), *Candida* spp. (n = 12), and *E. faecalis* (n = 7). Among the blood culture results, four cases showed methicillin-resistant *S. aureus* and ESBL-producing *E. coli.* All bacteraemia-associated *S. aureus* (n = 4) strains were methicillin-resistant and *E. coli* (n = 9) starins were ESBL-producing. There was no carbapenem-resistant bacterial infection in the study cohort.

During the FEN + COVID-19 episode, a total of 16 confirmed fungal infections, two probable Aspergillosis, and 22 possible Aspergillosis cases developed in the study group. Five of the fungal coinfections (2 proven Aspergillosis, 1 proven *Candida*, 2 probable Aspergillosis) had fungal pneumonia. Aspergillosis (including the possible and probable Aspergillosis cases) was the most common coinfection. Table 3 summarizes the overall causative agents in the MDI subgroup and the distribution of the CDI.

**Table 3:** Distribution of etiological agents versus microbiologically defined infections and clinically defined infections.

Infection	Microorganism	n (%)
Bacteraemia (n:34)	*E.coli*	9 (26.4%)
	*Acinetobacter spp.*	4 (11.7%)
	*S. aureus*	4 (11.7%)
	*Enterococcus spp.*	5 (14.7%)
	*S. pneumoniae*	4 (11.7%)
	*CoNS*	4 (13.5%)
	*Pseudomonas spp*	3 (8.8%)
	*Proteus mirabilis*	1 (2.9%)
Urinary tract infection (n:23)	*Candida spp.*	12 (46.1%)
	*E. coli*	5 (19.2%)
	*Enterococcus spp.*	5 (26.9%)
	*Pseudomonas spp.*	1 (3.8%)
Pneumonia (n:15)	*Stenotrophomonas maltophilia*	4 (26.6%)
	*E.coli*	2 (13.3%)
	*Acinetobacter spp.*	2 (13.3%)
	*S. aureus*	2 (13.3%)
	*S. pneumoniae*	2 (13.3%)
	*Aspergillus spp.*	2 (13.3%)
	*Candida spp.*	1 (6.6%)
Proven sinusitis	*Aspergillus flavus*	1(100%)
Probable *Aspergillus* pneumonia		*2*
Possible aspergillosis		*22*
3rd generation cephalosporin-resistant Gram-negative (n:10)	*E. coli*	4 (40%)
	*K. pneumoniae*	1 (10%)
	*Enterobacter cloacae*	1 (10%)
	*Proteus mirabilis*	1 (10%)
	*Pseudomonas spp.*	1 (10%)
	*Stenotrophomonas maltophilia*	2 (20%)
Methicillin-resistant staphylococci (n:11)	Coagulase negative *staphylococci*	7 (63.6%)
	*S.aureus*	4 (36.4%)
CDI (n:61)	Skin and soft tissue infection	40 (65.6%)
	Pneumonia	21 (34.4%)

*CDI* clinically documented infection.

Significant values are in (italic).

### Empirical antibiotic treatment for febrile neutropenia

In total, 11.2% (19/170) of the cohort received carbapenem-based antibacterial monotherapy, 35.9% (61/170) received beta lactam/beta lactamase inhibitor (BL/BLI) monotherapy. Glycopeptide-including combination empirical therapy was administered to 43.5% (74/170) of the patients, while 18.2% (31/170) received a combination of quinolone/macrolide as empirical therapy. Lastly, 13.5% (23/170) of the patients received antifungal agents as part of their empirical antimicrobial treatment (Table 1).

### Analysis of the overall cohort according to MASCC score

We analysed the main study parameters to compare the low-risk and high-risk FEN patients. Our results revealed that the following factors were significantly higher in the high-risk FEN group: no recovery from neutropenia at any time during treatment, duration of recovery from neutropenia, any fungal infection, including possible, probable, and definite fungal infections, possible + probable + proven aspergillosis, proven + probable aspergillosis, any possible fungal infection, age ≥ 60, having d-dimer ≥ 1000 µg/L, neutrophil count below 250/mm^3^, CRP levels ≥ 100 mg/L, having an underlying haematological disease, receiving empirical antifungal therapy, receiving empirical glycopeptide therapy at any time during the COVID-19/FEN episode, receiving intravenous immunoglobulin (IVIG), receiving carbapenem therapy including anytime during the COVID-19/FEN episode, need for mechanical ventilation, septic shock during the COVID-19/FEN episode, need for ICU during the COVID-19/FEN episode and one-month mortality. Please refer to (Table 2S in supplementary material) for details of all parameters including non-significant parameters.

### COVID-19 symptoms and oxygen support

The three most frequently reported symptoms other than fever, on the day of COVID-19/FEN were cough (55.9%), shortness of breath (25.9%), and headache (23.5%), respectively. Overall, 16 (9.4%) were asymptomatic except for fever on the day of COVID-19 diagnosis. The distribution of symptoms in the study cohort is summarized in Table 1.

Overall, 84.1% (143/170) of the cases were on room air on the day of COVID-19 diagnosis. During follow-up, 41.6% (81/170) and 25.9% (44/170) cases required supplemental oxygen and mechanical ventilation, respectively. Additionally, 34 (20%) cases developed septic shock during the course of COVID-19/FEN.

### COVID-19 associated treatment and viral clearance

The three most commonly used COVID-19-associated treatments were favipiravir (94.1%-160/170), plasma (12.5%-20/170), and IV immunoglobulin (12.9%-22/170) (Table 4).

**Table 4:** Summary of antiviral treatment modalities versus end of treatment PCR negativity.

Treatment	End of treatment COVID-19 negative (N: 71)	End of treatment COVID-19 positive (N: 19)	*p*
Any favipiravir	68 (95.7%)	17 (89.5%)	0.286
No favipiravir	3 (4.3%)	2 (10.5%)	
Up to 10 day favipiravir	6 (8.5%)	7 (36.9%)	0.002
Up to 5 day favipiravir	62 (87.3%)	10 (52.6%)	
No favipiravir	3 (4.2%)	2 (10.5%)	
Up to 10 day favipiravir	6 (8.5%)	7 (36.9%)	< 0.001
Up to 5 day favipiravir	62 (87.3%)	10 (52.6%)	
Up to 5 day favipiravir	62 (87.3%)	10 (52.6%)	0.119
No favipiravir	3 (4.2%)	2 (10.5%)	
Up to 10 day favipiravir	6 (8.5%)	7 (36.9%)	0.598
No favipiravir	3 (4.2%)	2 (10.5%)	
COVID-19 convalescent plasma	12 (16.9%)	0 (0%)	0.054
No plasma	59 (83.1%)	19 (100%)	
IV immunoglobulin	4 (5.6%)	4 (21.1%)	0.035
No IV immunoglobulin	67 (94.4%)	15 (78.9%)	
Hydroxychloroquine	6 (8.5%)	2 (10.5%)	0.777
No hydroxychloroquine	65 (91.5%)	17 (89.5%)	
Famotidine	11 (15.5%)	5 (26.3%)	0.273
No famotidine	60 (84.5%)	14 (73.7%)	
Remdesivir	0 (0%)	1 (5.3%)	NC
No remdesivir	71 (100%)	18 (94.7%)	

*NC* not calculated due to low n.

Viral clearance during the treatment of COVID-19 was evaluated in a total of 94 cases. The rates of viral clearance at 48–72 h and at the EOT were 19.7% (14/71) and 78.9% (71/90), respectively. In the univariate analysis for PCR negativity, there was a significant difference between treatments with up to 5 days of favipiravir vs. up to 10 days favipiravir vs. no favipiravir (*p* = 0.002). Also, there was a significant difference between up to 5 days of favipiravir vs. up to 10 days of favipiravir (*p* < 0.001). Table 4 presents the overall data and the results of the univariate analysis for favipiravir, plasma therapy, IVIG therapy, hydroxychloroquine, famotidine, and remdesivir in relation to viral clearance (all *p* > 0.05).

### Univariate analysis of the overall cohort according to need for ICU during the COVID-19/FEN episode

Comparison of patients who required ICU admission vs. others during the COVID-19/FEN episode revealed the following factors to be significantly more prevalent in the cohort requiring ICU: chronic renal failure, lack of recovery from neutropenia, being classified as high risk for FEN (MASCC < 21), mean LDH levels, LDH levels ≥ 250 U/L, ferritin levels ≥ 2000 µg/L, d-dimer levels ≥ 1000 µg/L, d-dimer levels ≥ 2250 µg/L, initial neutrophil count ≤ 250/mm^3^, higher mean CRP levels, CRP levels ≥ 75 mg/L, CRP levels ≥ 100 mg/L, remaining COVID-19 PCR-positive during COVID-19/FEN episode, receiving any steroid treatment during the COVID-19/FEN episode, receiving tocilizumab therapy, receiving remdesivir therapy, receiving quinolone therapy at any time during the COVID-19/FEN episode, receiving glycopeptide therapy at any time during the COVID-19/FEN episode, receiving carbapenem therapy at any time during the COVID-19/FEN episode, need for supplemental oxygen, need for mechanical ventilation, developing septic shock during the COVID-19/FEN episode, and OMM. Details of all univariates including non-significant parameters are summarized in (Table 3S in supplementary material).

### Univariate analysis of the overall cohort according to need for mechanical ventilation during the COVID-19/FEN episode

When we compared the subgroups requiring mechanical ventilation during the COVID-19/FEN episode versus others, the following variables were significantly higher in the mechanical ventilation cohort; chronic renal failure, being high risk FEN (MASCC < 21), lack of recovery from neutropenia, higher mean LDH value, LDH levels ≥ 250 U/L, ferritin levels ≥ 2000 µg/L, having d-dimer ≥ 1000 µg/L, having d-dimer ≥ 2250 µg/L, mean initial neutrophil count, initial neutrophil count below 250/mm^3^, having CRP ≥ 75 mg/L, having CRP ≥ 100 mg/L, having an underlying haematological malignancy, receiving tocilizumab therapy, receiving remdesivir therapy, receiving carbapenem including empirical monotherapy, receiving empirical combination therapy with quinolones, receiving glycopeptide including therapy at anytime during the COVID-19/FEN episode, receiving IVIG, need for supplemental oxygen, developing septic shock during the COVID-19/FEN episode, the need for ICU during the COVID-19/FEN episode, and OMM. Detailed data related to all significant and non-significant parameters are summarized in (Table 4S in supplementary material).

### Mortality and Univariate analysis of the overall cohort according to one-month mortality

Our study cohort’s EOT, OMM and day 90 mortality rates were 35.9% (71/170), 44.8% (76/170) and 47.6% (81/170), respectively. EOT, OMM and day 90 mortality did not differ significantly according to the regions of Turkey (*p* > 0.05, Table 1S).

We performed a detailed univariate analysis for OMM. The following variables showed a significant association with OMM (Table 5): high risk FEN (*p* < 0.001), development of septic shock during follow-up (*p* < 0.001), need for ICU during follow up (*p* < 0.001), requirement of supplemental oxygen during follow-up (*p* < 0.001), need for mechanical ventilation during follow-up (*p* < 0.001), mean initial CRP level (*p* = 0.010), initial CRP ≥ 75 mg/L (*p* < 0.001), initial CRP ≥ 100 mg/L (*p* = 0.001), mean initial d-dimer level (*p* = 0.015), initial d-dimer ≥ 1000 µg/L (*p* = 0.039), mean initial LDH level (*p* = 0.031), mean initial ferritin level (*p* = 0.013), mean initial neutrophil count (*p* = 0.026), receiving IVIG treatment (*p* = 0.017), presence of chronic renal failure (*p* = 0.002), and use of carbapenem including therapy anytime during the COVID-19/FEN episode (*p* = 0.003). It is worth mentioning that out of 76 cases with OMM, 40 (52.6%) died after recovering from neutropenia. Table 5S (supplementary documents) contains overall parameters that were not associated with OMM.

**Table 5:** Univariate analysis for one-month mortality: significant variable.

Parameter	Day 30 survival N: 94	Day 30 mortality N: 76	*p*
IVIG			
Yes	7 (7.5%)	15 (19.7%)	0.017
No	87 (92.5%)	61 (80.3%)	
Carbapenem including therapy anytime during the COVID-19/FEN episode			
Yes	50 (53.2%)	57 (75%)	0.003
No	44 (46.8%)	19 (25%)	
Supplemental oxygen			
Yes	60 (63.8%)	67 (88.2%)	< 0.001
No	34 (36.2%)	9 (11.8%)	
Mechanical ventilation			
Yes	2 (2.1%)	42 (55.3%)	< 0.001
No	92 (97.9%)	34 (44.7%)	
Septic shock during COVID-19/FEN			
Yes	5 (5.3%)	29 (38.2%)	< 0.001
No	89 (94.7%)	47 (61.8%)	
Need for ICU during COVID-19/FEN			
Yes	12 (12.8%)	47 (61.8%)	< 0.001
No	82 (87.2%)	29 (38.2%)	
Chronic renal failure			
Yes	0 (0%)	7 (9.2%)	0.002
No	94 (100%)	69 (90.8%)	
Neutrophil (/mm^3^)	296 ± 205	230 ± 172	0.026
CRP (mg/L)	89 ± 118	130 ± 90	0.013
CRP ≥ 75 mg/L	50 (53.2%)	58 (76.3%)	< 0.001
CRP < 75 mg/L	44 (46.8%)	18 (23.7%)	
CRP ≥ 100 mg/L	38 (40.4%)	50 (65.8%)	0.001
CRP < 100 mg/L	56 (59.4%)	26 (34.2%)	
D-dimer (µg/L)	1355 ± 1729	2823 ± 5479	0.015
D-dimer < 1000 µg/L	54 (60.7%)	30 (44.1%)	0.039
D-dimer ≥ 1000 µg/L	35 (39.3%)	38 (55.9%)	
Ferritin (µg/L)	1124 ± 1658	2754 ± 6091	0.013
LDH (U/L)	307 ± 213	412 ± 405	0.031
High risk FEN MASCC (< 21)	28 (29.8%)	43 (56.6%)	< 0.001
Low risk FEN MASCC (≥ 21)	66 (70.2%)	33 (43.4%)	

*IVIG* intravenous immunoglobulin, *FEN* febrile neutropenia, *ICU* intensive care unit, *CRP* C-reactive protein, *LDH* lactate dehydrogenase, *MASCC* Multinational Association of Supportive Care in Cancer.

Tocilizumab treatment, compared to no tocilizumab treatment, did not show an association with mortality in the overall cohort (*p* = 0.173, supplementary documents). However, it was found to be associated with lower mortality in the subgroup with CRP ≥ 75 mg/L compared to others (1/7 vs. 57/101; *p* = 0.030).

## Multivariate analysis

### PCR negativity

Logistic regression analysis showed that receiving up to 5 days of favipiravir (*p* = 0.005, OR 5.166, 95% CI 1.639–16.280) was significantly linked to achieving PCR negativity during the COVID-19 and febrile neutropenia episode (see Table 6S in the supplemental material).

#### Need for ICU during the COVID-19/FEN episode

The logistic regression analysis for the need for ICU during the COVID-19/FEN episode showed the following significant variables: receiving glycopeptide therapy at any time during the COVID-19/FEN episode (*p* = 0.001, OR 6.566, 95% CI 2.137–20.172) and the need for mechanical ventilation (*p* < 0.001, OR 62.042, 95% CI 9.528–404.011). The complete list of significant and non-significant variables can be found in (Table 7S, supplemental material).

#### Need for mechanical ventilation during the COVID-19/FEN episode

The logistic regression analysis for the need for mechanical ventilation during the COVID-19/FEN episode revealed the following variables significant: failure to recover from neutropenia (*p* < 0.001, OR 17.869, 95% CI 3.592–88.907), receiving tocilizumab therapy (p = 0.028, OR 32.227, 95% CI 1.469–707.053), development of septic shock during the COVID-19/FEN episode (*p* = 0.001, OR 15.4 96% CI 3.164–75.897), and the need for ICU during the COVID-19/FEN episode (*p* < 0.001, OR 91.818, 95% CI 15.360–548.873). For a complete list of significant and non-significant variables, please refer to (Table 8S, supplemental material).

#### One-month mortality

Logistic regression analysis for OMM revealed that mechanical ventilation (*p* ≤ 0.001, OR 22.904, 95% CI 4.211–124.573) and septic shock (*p* = 0.012, OR 5.125, 95% CI 1.435–18.306) were significantly associated with OMM. All significant and non-significant variables are shown in (Table 9S, supplemental material).

#### Reinfections

There were 5 (6.2% of 81 day 90 survivors, 2.9% of the overall cohort) COVID-19 reinfections during 90-day follow up (until day 180 of COVID-19/FEN episode).

## Discussion

Despite advancements in modern medicine, FEN remains a significant cause of illness and death in cancer patients^7,8,10,13^. Neutropenia commonly arises as a result of cancer chemotherapy. Patients are highly susceptible to bacterial, viral, and fungal infections during the neutropenic phase. Although the mortality rates are approximately 5% in patients with solid tumours and 11% in haematological malignancies, this percentage can rise up to 50% in hospitalized patients^4^.

While the mortality rate of COVID-19 in the global population of all patients was 1.02% (6,656,601/651,918,402) as of the deadline of this study, the risk of mortality is significantly higher among individuals with weakened immune systems, including those with malignancies^2^. Studies have shown that the mortality rate of COVID-19 among patients with malignancies ranges from 15 to 61%^14–17^.

Data regarding COVID-19, mortality, and its influencing factors in patients with FEN are extremely limited. According to a recent systematic review involving 19 patients, the overall mortality rate in this group was 15.8%, whereas it reached 27% in individuals with haematological malignancies (n = 11)^14^. No fatality was observed in patients without malignancies or solid organ tumours^14^. A yet unpublished ICD (international classification of diseases) code based study conducted in United States by Fatuyi et al.^18^, showed that the concurrent presence of COVID-19 in adult patients hospitalized with FEN increased the mortality rate by 14 times (OR 13.6, 95% CI 3.6–51.8). In our study, the rate of OMM was quite high (44.8%) and did not show significant variation between haematological and solid malignancies. Interestingly, probably COVID-19-associated neutropenia and FEN accounted for 47.4% of OMM in our cohort.

The requirement for mechanical ventilation during the course of COVID-19 is a crucial factor linked to mortality. According to a systematic review of 12,437 cases, it was found that 43–74% of COVID-19 patients who required invasive mechanical ventilators had mortality during the follow-up^19^. Ceken et al.^20^ examined the factors linked to mortality in a series of 135 FEN cases and reported that the requirement for mechanical ventilation was a significant risk factor associated with increased mortality (OR 5.36, *p* 0.0001 CI 1.53–18.80). Fatuyi et al.^18^ reported that the occurrence of respiratory failure in patients with FEN and COVID-19 was 11 times higher compared to FEN patients without COVID-19. Another study examined neutropenic oncology patients undergoing active chemotherapy along with COVID-19, and reported that 63.4% of the 83 patients experienced respiratory failure. Among them, 37.8% required high FiO_2_ (> 35%) support^21^. In our series, 74.7% of the patients needed oxygen support and 25.8% required mechanical ventilation. It is possible that this difference is due to the inclusion of patients with haematological malignancies and without any malignancies in our patient group. In our series, the OMM was extremely high (95.4%) among patients who required mechanical ventilation during the COVID-19 and FEN episodes. Furthermore, our univariate and multivariate analysis indicated that (i) as expected the requirement for ICU (*p* < 0.001, OR 91.818, 95% CI 15.360–548.873) was associated with the necessity of mechanical ventilation (ii) the need for mechanical ventilation in the follow-up of patients with COVID-19 and FEN was associated with a significant increase in OMM (*p* = 0.001, OR 19.041, 95% CI 3.229–112.286). Finally, we believe that the significant association between the need for ICU and the use of glycopeptide therapy at any point during the COVID-19/FEN episode (*p* = 0.001, OR 6.566, 95% CI 2.137–20.172) in the logistic regression analysis is probably due to the recommendation of glycopeptide, including combination therapy, in severe pneumonia as suggested by the International Data Spaces Association (IDSA) FEN guidelines^4^.

Septic shock is universally linked with higher rates of mortality and morbidity^18,22,23^. According to a meta-analysis, the 30-day mortality rate for patients with septic shock was found to be 33.7% in the USA, 32.5% in Europe, and 26.4% in Austria^22^. In a multicentre study conducted in Turkey, the in-hospital mortality rate was reported as 72.6%^23^. It has also been reported that septic shock is associated with significantly higher rate of mortality in patients with FEN^22^. Fatuyi et al.^18^ (also mentioned above), analysed 16,790 patients with FEN. They found that the risk of developing septic shock was 21 times higher in 145 patients with concurrent COVID-19 compared to those without COVID-19^18^. Additionally, they showed that septic shock was a significant factor in in-hospital mortality for patients with FEN and COVID-19 infection [HR 9.2; 95% CI 5.8–13.6; *p* < 0.05]^18^. Consistent with the literature^18,22^, in our study, the overall mortality rate was very high (84.8%) in the subgroup of septic shock patients. Our findings also demonstrated that the development of septic shock during the follow-up period had a significant impact on the need for mechanical ventilation (*p* = 0.001, OR 15.4 96% CI 3.164–75.897) and OMM (*p* = 0.010, OR 5.589, 95% CI 1.509–20.700).

Tocilizumab, an interleukin-6 inhibiting monoclonal antibody, is suggested for use in the treatment of COVID-19, particularly in patients with macrophage activation syndrome. Based on the evaluation of randomized controlled trials involving 6481 patients, it was found that compared to those receiving standard therapy without tocilizumab, those given tocilizumab had a lower rate of all-cause mortality (RR 0.89; 95% CI 0.82–0.98) 21–28 days after starting treatment^24^. According to European Society of Clinical Microbiology and Infectious Diseases (ESCMID) guidelines, it is recommended to use tocilizumab in hospitalized patients with severe COVID-19^24^. A study conducted in cancer patients indicated that higher COVID-19 severity was associated with abnormal CRP levels^25^. Another randomized clinical trial on tocilizumab reported that patients with CRP levels above 150 mg/L benefited from tocilizumab treatment^26^. In the RECOVERY study, tocilizumab was shown to be an effective treatment option for patients with CRP levels > 75 mg/L^11^. In our study, the use of tocilizumab did not have a significant effect on OMM in the overall cohort. However, consistent with the existing literature, we observed a significant reduction in OMM in the subgroup of patients with a CRP value > 75 mg/L. Furthermore, the analysis of univariate and logistic regression revealed that the use of tocilizumab was associated with the need for mechanical ventilation (*p* = 0.028, OR 32.227, 95% CI 1.469–707.053). This association may be due to the very limited availability of tocilizumab in hospitals during the study period, leading to its prioritization for only the most severe cases.

There are multiple case reports in the literature^27–29^ that present severe neutropenia caused by COVID-19. This could explain why our study subgroup, who did not have any known malignancy also experienced neutropenic condition and FEN. Additionally, our logistic regression analysis resulted that patients who did not recover from neutropenia during the COVID-19/FEN episode had a significantly higher need for mechanical ventilation (*p* < 0.001, OR 17.869, 95% CI 3.592–88.907). The fact that 47.4% of patients in the COVID-19-associated neutropenia subgroup had OMM further reinforces the idea that persistent neutropenia is a negative prognostic indicator.

It is considered that the time to viral clearance during COVID-19 is closely related to the host immunity. A study that examined the rate of viral clearance found that on day 14, the clearance rate was 32%, and on day 28, it was 54%. Similar to our findings, the study did not find any association between viral clearance and mortality. However, they reported that the presence of concomitant neoplasia was linked to a decrease in viral clearance (HR 0.127; CI 95% 0.023–0.0702; *p* = 0.018)^30^. In another meta-analysis, a 7-day standard dose of favipiravir treatment was found to result in higher viral clearance compared to the comparator group (OR 2.49, 95% Cl 1.19–5.22)^31^. In our study, the negation of SARS-CoV-2 PCR had a non-significant effect (*p*: 0.105) on OMM in the entire cohort. However, our logistic regression analysis resulted that receiving up to 5 days of favipiravir treatment was associated with increased PCR negativity (*p* = 0.005, OR 5.166, 95% CI 1.639–16.280).

Opportunistic fungal infections are among common problems developing in FEN and are associated with increased mortality^4^. Moreover, COVID-19 patients may also experience fungal co-infections, particularly invasive aspergillosis infections. The presence of COVID-19-associated pulmonary aspergillosis was found to be associated with a longer period of mechanical ventilation and increased mortality^32,33^. Studies have also reported co-infections with candidiasis, mucormycosis, and cryptococcosis in COVID-19 patients^34^. In our study, 23.5% (40/170) of the overall cohort had a fungal co-infection. However, the presence of proven, possible, or probable fungal coinfections did not impact the 30-day mortality rate significantly. We did not identify any cases of mucormycosis or cryptococcosis in our cohort. This might be due to the relatively limited number of mycological culture samples that were sent for testing.

Patients with both COVID-19 and malignancies have a higher likelihood of needing ICU care compared to those without malignancy^35,36^. Several factors that may contribute to this condition have been reported in the literature, including male gender, chronic renal failure, severe COVID-19 symptoms, older age, and chronic obstructive pulmonary disease^37,38^. However, to our knowledge, there is no detailed published analysis in the literature that specifically examines the factors influencing the requirement for intensive care in patients with both COVID-19 and FEN. Brogan et al.^39^ reported that COVID-19 patients with chronic renal failure who were admitted to the ICU had a higher mortality rate. In our univariate analysis, we also found that having chronic renal failure was associated with the need for ICU, mechanical ventilation, and OMM (Table 5, Tables 4S, 5S). However, we were unable to include the variable of chronic renal failure in the logistic regression model for OMM and mechanical ventilation due to uneven frequency distribution (supplementary material). Additionally, the logistic regression analysis model for the need for ICU did not show a significant odds ratio for chronic renal failure (*p* = 0.735, OR 0.841, 95% CI 0.036–15.005), which might be attributed to the relatively small number of patients with chronic renal failure in our cohort (n = 7).

It is well established that a MASCC score below 21 is associated with a higher risk of mortality in FEN^4^. Additionally, in COVID-19 patients, factors such as lymphopenia, thrombocytopenia, elevated d-dimer levels, and high CRP are associated with a poor prognosis and increased mortality^12^. Development of oxygen demand and needing intensive care during clinical follow-up are also crucial indicators of a negative COVID-19 prognosis. In a study involving patients with oncological malignancy and COVID-19, it was found that low neutrophil levels, high LDH levels, and high oxygen demand were associated with a significantly higher mortality rate^21^. In our study, we found that several factors were effective on OMM or need for ICU care or MV in univariate analysis. These factors included: MASCC score (≥ 21 vs. < 21), IVIG therapy, treatment regimen including carbapenem, need for intensive care, oxygen support, CRP level (CRP ≥ 75 mg/L vs. CRP < 75 mg/L, CRP ≥ 100 mg/L vs. CRP < 100 mg/L) initial neutrophil count (≥ 250/mm^3^ vs. < 250/mm^3^), d-dimer (≥ 1000 µg/L vs. < 1000 µg/L) level, ferritin level, and LDH level parameters. However, logistic regression analysis did not reveal any of these variables (including being high risk FEN according to the initial MASCC score) to be associated with OMM. It is possible to speculate that the absence of a significant correlation between high risk FEN (low MASCC score) or neutrophil recovery vs. OMM in the multivariate analysis could be attributed to: (i) out of 76 cases, 40 (52.6%) who had OMM, died after recovering from neutropenia, and (ii) the MASCC score reflects the clinical presentation on day 1; the MASCC score might have decreased not on day 1 of the COVID-19/FEN episode, but in the subsequent days.

In the presented study, we utilized logistic regression analysis as the method for multivariate analysis. Logistic regression is commonly used for binary events, such as mortality or other parameters examined in our study. Logistic regression may include only one or multiple independent variables, although examining multiple variables is generally more informative because it reveals the unique contribution of each variable after adjusting for the others. As in our study, OMM may be associated with many univariate variables. Logistic regression analysis aids in assessing multiple independent variables simultaneously, rather than looking at each variable in isolation. It is considered that if the logistic regression model includes multiple independent variables, the ORs are now “adjusted” because they represent the unique contribution of the independent variable after adjusting for (or subtracting out) the effects of the other variables in the model^40^. It is also important to mention that when analyzing the variables related to antibacterial therapy and the inflammatory COVID-19 markers (CRP, d-dimer, lactate dehydrogenase, ferritin), as they showed a correlation, we incorporated into the logistic regression models the variables with the lowest *p*-value^40^.

Our study has several limitations. Although it was a multicentre study, this was a retrospective cohort study held only in one country. There was no control/comparator group compared with non-COVID-19 FEN cases. Another limitation is the fact that only COVID-19 PCR positive cases were included. We are aware that certain COVID-19 cases may test negative for PCR due to mutations in the COVID-19 genome^41^. Viral culture was not performed in any case. In all MDI cases, negative outcomes could also be attributed to coinfection. However, we were unable to perform autopsies or analyse infection-related mortality. Instead, we relied on 1-month survival data as a measure of all-cause mortality. Therapeutic drug monitoring for vancomycin, voriconazole, etc. was not conducted in any of the cases Due to the limited number of reinfections (only 5), it was not possible to perform a detailed statistical analysis or regression analysis. Nevertheless, to our knowledge, this is the first study to analyse COVID-19 and FEN with a quite extended scope and analysis (viral clearance, high and low risk FEN, need for ICU, need for mechanical ventilation and OMM) in a relatively large patient group. It also provides valuable data regarding outcomes of basic COVID-19 treatment options in FEN cases.

In summary, our analysis, which includes a relatively small yet noteworthy number of patients, provides insights into the progression of COVID-19, available treatment options, and survival rates in cases of adult FEN and COVID-19. These findings indicate that COVID-19 and FEN are linked to substantial OMM. Additionally, according to the logistic regression analysis results, the following variables had a positive influence on the mentioned dependent variables: (i) achieving PCR negativity: receiving a maximum of 5 days of favipiravir (*p* = 0.005, OR 5.166, 95% CI 1.639–16.280); (ii) need for ICU: receiving glycopeptide therapy at any time during the COVID-19/FEN episode (*p* = 0.001, OR 6.566, 95% CI 2.137–20.172), the need for mechanical ventilation (*p* < 0.001, OR 62.042, 95% CI 9.528–404.011); (iii) need for mechanical ventilation: failure to recover from neutropenia (*p* < 0.001, OR 17.869, 95% CI 3.592–88.907), receiving tocilizumab therapy (*p* = 0.028, OR 32.227, 95% CI 1.469–707.053), septic shock (*p* = 0.001, OR 15.4 96% CI 3.164–75.897), and the need for ICU (*p* < 0.001, OR 91.818, 95% CI 15.360–548.873), iv)OMM: [mechanical ventilation (*p* = 0.001, OR 19.041, 95%CI 3.229–112.286) and septic shock (*p* = 0.010, OR 5.589, 95% CI 1.509–20.700)].

### Supplementary Information

Supplementary Information.

## Data Availability

The data that support the findings of this study are available on upon reasonable request from the corresponding author with a research protocol and ethical approval.
