# Supplementary material for: The clinical features, treatment and prognosis of neutropenic fever and Coronavirus disease 2019 results of the multicentre teos study
Source: Sci Rep. 2024 Mar 3;14:5218. doi: 10.1038/s41598-024-55886-w (PMC10909849; doi:10.1038/s41598-024-55886-w)
Supplement: Supplementary file 1 — Supplementary Information. [file 41598_2024_55886_MOESM1_ESM.docx]

**Supplementary material**

**Methods**

**Logistic regression analysis of day 30 mortality:**

i) We did not include the variable chronic renal failure in the logistic regression model because its frequencies were not equally distributed.

ii) When considering the inflammatory COVID-19 markers (CRP, d-dimer, lactate dehydrogenase, ferritin), since they were correlating parameters, we included in the model the one with the lowest p value [40].

iii) Although the mean neutrophil count was significantly lower in the day 30 mortality group vs. others, we excluded it from the analysis. This decision was made because the two cut-off values we used in our study (neutrophil count <250/mm^3^ or <100/mm^3^) did not show a significant result.

**Logistic regression analysis of PCR negativity:**

We did not include the variable "receiving up to 10 days of favipiravir" in the logistic regression model because it was associated with receiving 5 days of favipiravir. In clinical practice, the favipiravir treatment was extended up to 10 days when there was no viral clearance on day 5.

**Logistic regression analysis of need for intensive care unit (ICU):**

i) When considering the inflammatory COVID-19 markers (CRP, d-dimer, lactate dehydrogenase, ferritin), since they were correlating parameters, we included in the model the one with the lowest p value [40].

ii) We did not include the variable receiving remdesivir in the logistic regression model because its frequencies were not equally distributed.

iii) When considering the antibacterial therapy related variables, since they were correlating parameters, we included in the model the one with the lowest p value [40].

**Logistic regression analysis of need for mechanical ventilation during the COVID-19/FEN episode:**

i) When considering the inflammatory COVID-19 markers (CRP, d-dimer, lactate dehydrogenase, ferritin), since they were correlating parameters, we included in the model the one with the lowest p value [40].

ii) We did not include the variables chronic renal failure, need for supplementary oxygen receiving tocilizumab and receiving remdesivir in the logistic regression model because their frequencies were not equally distributed.

iii) When considering the antibacterial therapy related variables, since they were correlating parameters, we included in the model the one with the lowest p value [40].

Table 1S End of treatment, day 30 and day 90 mortality according to the regions of Turkey

| Regions of Turkey | Marmara  (n=50) | Central Anatolia (n=46) | Southeastern Anatolia  (n=19) | Aegean  (n=18) | Eastern Anatolia  (n=14) | Mediterrenean  (n=12) | Black sea  (n=11) | p |
| --- | --- | --- | --- | --- | --- | --- | --- | --- |
| End of treatment  mortality  (61/170-35.9%) | 17  (27.9%) | 15  (24.6%) | 9  (14.8%) | 5  (8.2%) | 6  (9.8%) | 6  (9.8%) | 3  (4.9%) | 0.732 |
| One month mortality  (76/170-44.7%) | 17  (22.4%) | 19  (25%) | 11  (14.5%) | 10  (13.1%) | 7  (9.2%) | 7  (9.2%) | 5  (6.6%) | 0.450 |
| Day 90 mortality  (81/170-47.6%) | 18  (22.2%) | 22  (27.2%) | 11  (13.6%) | 10  (12.3%) | 8  (9.9%) | 7  (8.6%) | 5  (6.2%) | 0.537 |

Table 2S: Univariate analysis for high and low risks febrile neutropenia

| Parameter | | High risk  MASCC <21  N: 71 | Low risk  MASCC ≥21  N: 99 | p |
| --- | --- | --- | --- | --- |
| Female | | 27 (46.5%) | 31 (53.5%) | 0.362 |
| Age | | 60.5 ± 15.2 | 58.4± 15.8 | 0.386 |
| Chronic renal failure | | Present 5 (34.2 %)  Absent 66 (58.5%) | Present 2 (65.8%)  Absent 89 (41.5%) | 0.126 |
| Recovery from neutropenia during treatment | | Present 40 (34.2 %)  Absent 31 (58.5%) | Present 77 (65.8%)  Absent 22 (41.5%) | 0.002 |
| The mean time for recovery from neutropenia | | 6.6 ± 5.5 | 4.9± 3.1 | 0.011 |
| COVID-19 convalescent plasma | | Present 9 (45%)  Absent 62 (41.3%) | Present 11 (55%)  Absent 88 (58.7%) | 0.754 |
| Bacterial coinfection | | Present 29 (50.8%)  Absent 42 (37.2%) | Present 28 (49.2%)  Absent 71 (62.8%) | 0.087 |
| Fungal coinfection (proven+probable+possible) n:40 | | Present 28 (70%)  Absent 43 (33.1%) | Present 12 (30%)  Absent 87 (66.9%) | <0.001 |
| Aspergillosis (Proven+ Probable+possible) n:27 | | Present 21 (77.8%)  Absent 50 (34.9%) | Present 6 (22.2%)  Absent 93 (65.1%) | <0.001 |
| Aspergillosis (Proven+ Probable) n:5 | | Present 5 (100%)  Absent 66 (40%) | Present 0 (0%)  Absent 99 (60%) | 0.007 |
| Any proven fungal coinfection | | Present 10 (62.5%)  Absent 61 (39.6%) | Present 6 (37.5%)  Absent 93 (60.4%) | 0.077 |
| Any possible Fungal coinfection | | Present 16 (72.7%)  Absent 55 (37.2%) | Present 6 (27.3%)  Absent 93 (62.8%) | 0.001 |
| Age ≥60 | | Present 29 (30.2%)  Absent 42 (56.7%) | Present 67 (69.8%)  Absent 32 (43.3%) | 0.0005 |
| Initial lymphocyte (/mm^3^) | | 1402 ± 6224 | 597 ± 668 | 0.203 |
| Initial lymphocyte <800/mm^3^ | | 59 (41.8%) | 82 (58.2%) | 0.963 |
| Initial lymphocyte ≥800/mm^3^ | | 12 (41.3%) | 17 (58.7%) |  |
| Ferritin <500 µg/L | | 19 (35.2%) | 35 (64.8%) | 0.190 |
| Ferritin ≥500 µg/L | | 43 (46.2%) | 50 (53.8%) |  |
| LDH ≥250 U/L | | 34 (41.5%) | 48 (58.5%) | 0.834 |
| LDH< 250 U/L | | 34 (40.5%) | 50 (59.5%) |  |
| LDH (U/L) | | 351 ± 254 | 362 ± 358 | 0.827 |
| Ferritin (µg/L) | | 2120 ±2413 | 1986 ± 5490 | 0.847 |
| Ferritin <2000 µg/L | | 45 (39.5%) | 69 (60.5%) | 0.217 |
| Ferritin ≥2000 µg/L | | 17 (51.5%) | 16 (48.5%) |  |
| D-dimer (µg/L) | | 1925 ± 2869 | 2037 ± 4470 | 0.853 |
| D-dimer <1000 µg/L | | 25 (29.8%) | 59 (70.2%) | 0.032 |
| D-dimer ≥1000 µg/L | | 39 (53.4%) | 34 (54.6%) |  |
| D-dimer <2250 µg/L | | 47 (37.6%) | 78 (62.4%) | 0.110 |
| D-dimer ≥2250 µg/L | | 17 (53.1%) | 15 (46.8%) |  |
| Neutrophil (/mm^3^) | | 217 ± 167 | 293 ± 177 | 0.005 |
| Initial Neutrophil≥250/mm^3^ | | 29 (32.2%) | 61 (67.8%) | 0.007 |
| Initial Neutrophil<250/mm^3^ | | 42 (52.5%) | 38(47.5%) |  |
| CRP (mg/L) | | 128 ± 84 | 112 ± 123 | 0.344 |
| CRP ≥75 mg/L | | 50 (46.3%) | 58 (53.7%) | 0.113 |
| CRP <75 mg/L | | 21 (33.9%) | 41 (66.1%) |  |
| CRP ≥100 mg/L | | 44 (50%) | 44 (50%) | 0.024 |
| CRP <100 mg/L | | 27 (32.9%) | 55 (67.1%) |  |
| Underlying any solid tumors | | 16 (27.6%) | 42 (72.4%) | 0.002 |
| Underlying hematologic malignity | | 45 (48.3%) | 48 (51.7%) |  |
| No malignity | | 10 (52.6%) | 9 (47.4%) |  |
| PCR negativity during COVID-19 treatment | | Present 26 (38.8%)  Absent 8 (26.7%) | Present 41 (61.2%)  Absent 22 (73.3%) | 0.246 |
| Any steroid treatment in the overall cohort | | 43 (38.1%) | 70 (61.9%) | 0.167 |
| No steroid treatment in the overall cohort | | 28 (49.1%) | 29 (50.9%) |  |
| Tocilizumab | | 6 (66.7%) | 3 (33.3%) | 0.119 |
| No tocilizumab | | 65 (%) | 96 (%) |  |
| Favipiravir | | Present 65 (40.6%)  Absent 6 (60%) | Present 95 (59.4%)  Absent 4 (40%) | 0.228 |
| Remdesivir | | 2 (66.7%) | 1 (33.3%) | 0.377 |
| No remdesivir | | 69 (41.3%) | 98 (58.7%) |  |
| Hydroxychloroquine | | 9 (%50) | 9 (%50) | 0.453 |
| No hydroxychloroquine | | 62 (40.8%) | 90 (59.2%) |  |
| Famotidine | | 13 (52%) | 12 (48%) | 0.369 |
| No famotidine | | 64 (42.4%) | 87 (57.6%) |  |
| Colchicine | | 0 (0%) | 3 (100%) | 0.138 |
| No colchicine | | 71 (42.5%) | 96 (57.5%) |  |
| BL/BLI including empirical monotherapy | Present 20 (32.8%)  Absent 51 (46.8%) | Present 41 (67.2%)  Absent 58 (53.2%) | 0.075 |  |
| Carbapenem including empirical monotherapy | Present 9 (47.4%)  Absent 62 (41.1%) | Present 10 (52.6%)  Absent 89 (58.9%) | 0.599 |  |
| Empirical combination therapy with quinolones | Present 2 (18.2%)  Absent 69 (43.4%) | Present 9 (81.8%)  Absent 90 (56.6%) | 0.101 |  |
| Antifungal including empirical therapy | Present 18 (78.3%)  Absent 53 (36.1%) | Present 5 (21.7%)  Absent 94 (63.9%) | <0.001 |  |
| Glycopeptide including therapy anytime during the COVID-19/FEN episode | Present 54 (27.1%)  Absent 17 (17.7%) | Present 20 (72.9%)  Absent 79 (82.3%) | <0.001 |  |
| BL/BLI including therapy anytime during the COVID-19/FEN episode | | Present 38 (40.9%)  Absent 33 (42.8%) | Present 55 (59.1%)  Absent 44 (57.2%) | 0.792 |
| Fluoroquinolone or macrolide including therapy anytime during the COVID-19/FEN episode | | Present 16 (51.6%)  Absent 55 (39.6%) | Present 15 (48.3%)  Absent 84 (60.4%) | 0.218 |
| Additional CDI | | Present 21 (34.4%)  Absent 50 (45.8%) | Present 40 (65.6%)  Absent 59 (54.2%) | 0.146 |
| Receiving two doses of COVID-19 vaccine before COVID-19 episode | | Present 3 (27.3%)  Absent 68 (42.8%) | Present 8 (72.7%)  Absent 91 (57.2%) | 0.313 |

| IVIG | Present 14 (63.6%)  Absent 57 (38.5%) | Present 8 (36.4%)  Absent 91 (61.5%) | 0.025 |
| --- | --- | --- | --- |
| Carbapenem including therapy anytime during the COVID-19/FEN episode | Present 51 (47.7%)  Absent 20 (31.7%) | Present 56 (52.3%)  Absent 43 (68.3%) | 0.042 |
| Supplemental oxygen | Present 55 (43.3%)  Absent 16 (37.2%) | Present 72 (56.7%)  Absent 27 (62.8%) | 0.483 |
| Mechanical  ventilation | Present 30 (68.2%)  Absent 41 (32.5%) | Present 14 (31.8%)  Absent 85 (67.5%) | <0.001 |
| Septic shock during COVID-19/FEN | Present 20 (58.8%)  Absent 51 (37.5%) | Present 14 (41.2%)  Absent 85 (62.5%) | 0.024 |
| Need for ICU during COVID-19/FEN | Present 36 (61.1%)  Absent 35 (31.5%) | Present 23 (38.9%)  Absent 76 (68.5%) | <0.001 |
| One month mortality | Present 43 (56.6%)  Absent 28 (29.8%) | Present 33 (43.4%)  Absent 66 (70.2%) | <0.001 |
| Reinfection | Present 0 (0%)  Absent 71 (43%) | Present 5 (100%)  Absent 94 (57%) | 0.054 |
|  |  |  |  |
|  |  |  |  |

(PCR: polymerase chain reaction, BL/BLI: beta lactam beta lactamase inhibitor, FEN: febrile neutropenia, CDI: clinically documented infection, IVIG: intravenous immunoglobulin, ICU: intensive care unit, LDH: Lactic dehydrogenase, CRP: C reactive protein, COVID-19: Coronavirus disease 2019)

Table 3S: Univariate analysis for the need for intensive care unit admission

| Parameter | | ICU need  N: 59 | No ICU need  N: 111 | p |
| --- | --- | --- | --- | --- |
| Female | | 17 (29.3%) | 41 (70.7%) | 0.287 |
| Age | | 58.6 ± 15.4 | 58.8 ± 15.9 | 0.937 |
| Chronic renal failure | | Present 6 (85.7%)  Absent 53 (32.5%) | Present 1 (14.3%)  Absent 110 (67.5%) | 0.004 |
| No recovery from neutropenia during treatment | | 29 (54.7%) | 24 (45.3%) | <0.001 |
| Recovery from neutropenia during treatment | | 30 (25.6%) | 87 (74.4%) |  |
| The mean time for recovery from neutropenia | | 6.3 ± 5.5 | 5 ± 3.5 | 0.062 |
| COVID-19 convalescent plasma | | Present 10 (50%)  Absent 49 (32.7%) | Present 10 (50%)  Absent 101 (67.3%) | 0.126 |
| Bacterial coinfection | | Present 21 (36.8%)  Absent 38 (33.6%) | Present 36 (63.2%)  Absent 75 (66.4%) | 0.677 |
| Fungal coinfection (proven+probable+possible) n:40 | | Present 17 (42.5%)  Absent 42 (32.3%) | Present 23 (57.5%)  Absent 88 (67.7%) | 0.236 |
| Aspergillosis (Proven+ Probable+possible) n:27 | | Present 11 (40.7%)  Absent 48 (33.5%) | Present 16 (59.3%)  Absent 95 (66.4%) | 0.472 |
| Aspergillosis (Proven+ Probable) n:5 | | Present 3 (60%)  Absent 56 (33.9%) | Present 2 (40%)  Absent 109 (66.1%) | 0.227 |
| Any proven fungal coinfection | | Present 7 (43.8%)  Absent 52 (33.8%) | Present 9 (56.2%)  Absent 102 (66.2%) | 0.553 |
| Possible Fungal coinfection | | Present 8 (36.4%)  Absent 51 (34.5%) | Present 14 (63.6%)  Absent 97 (65.5%) | 0.861 |
| Age ≥60 | | Present 32 (33.3%)  Absent 27 (36.5%) | Present 64 (66.7%)  Absent 47 (63.5%) | 0.668 |
| Initial lymphocyte (/mm^3^) | | 1310 ± 6787 | 610 ± 875 | 0.284 |
| Initial lymphocyte <800/mm^3^ | | 52 (36.9%) | 89 (63.1%) | 0.189 |
| Initial lymphocyte ≥800/mm^3^ | | 7 (24.1%) | 22 (75.9%) |  |
| Ferritin <500 µg/L | | 15 (27.8%) | 39 (72.2%) | 0.179 |
| Ferritin ≥500 µg/L | | 36 (38.7%) | 57 (61.3%) |  |
| LDH≥250 U/L | | 36 (43.9%) | 46 (56.1%) | 0.026 |
| LDH<250 U/L | | 23 (27.4%) | 61 (72.6%) |  |
| LDH (U/L) | | 439 ± 406 | 324 ± 256 | 0.026 |
| Ferritin (µg/L) | | 2527 ± 3401 | 1587 ± 4709 | 0.209 |
| Ferritin <2000 µg/L | | 28 (26.9%) | 76 (73.1%) | 0.002 |
| Ferritin ≥2000 µg/L | | 23 (53.5%) | 20 (46.5%) |  |
| D-dimer(µg/L) | | 2747 ± 4081 | 1698 ± 3901 | 0.117 |
| D-dimer <1000 µg/L | | 21 (25%) | 63 (75%) | 0.007 |
| D-dimer ≥1000 µg/L | | 33 (40.2%) | 40 (59.8%) |  |
| D-dimer <2250 µg/L | | 37 (29.6%) | 88 (70.4%) | 0.012 |
| D-dimer ≥2250 µg/L | | 17 (53.1%) | 15 (46.9%) |  |
| Neutrophil (/mm^3^) | | 228 ± 197 | 277 ± 167 | 0.089 |
| Initial Neutrophil ≥250/mm^3^ | | 23 (25.6%) | 67 (74.4%) | 0.007 |
| Initial Neutrophil <250/mm^3^ | | 36 (45%) | 44 (55%) |  |
| CRP (mg/L) | | 152 ± 118 | 105 ± 103 | 0.007 |
| CRP ≥75 mg/L | | 47 (43.5%) | 61 (56.5%) | 0.001 |
| CRP <75 mg/L | | 12 (19.3%) | 50 (80.7%) |  |
| CRP ≥100 mg/L | | 42 (47.7%) | 46 (52.3%) | <0.001 |
| CRP <100 mg/L | | 17 (20.7%) | 65 (79.3%) |  |
| Underlying any solid tumors | | 18 (31.1%) | 40 (68.9%) | 0.086 |
| Underlying hematologic malignity | | 38 (40.9%) | 55 (59.1%) |  |
| No malignity | | 3 (15.8%) | 16 (84.2%) |  |
| PCR negativity during COVID-19 treatment | | Present 9 (13.4%)  Absent 18 (60%) | Present 58 (86.6%)  Absent 12 (40%) | <0.001 |
| Any steroid treatment in the overall cohort | | 45 (39.8%) | 68 (60.2%) | 0.048 |
| No steroid treatment in the overall cohort | | 14 (24.6%) | 43 (75.4%) |  |
| Any steroid treatment in O_2_ receiving cohort | | 44 | 51 | 0.575 |
| No steroid treatment in O_2_ receiving cohort | | 13 | 19 |  |
| Tocilizumab | | 6 (66.7%) | 3 (33.3%) | 0.038 |
| No tocilizumab | | 53 (32.9%) | 108 (67.1%) |  |
| Favipiravir | | 57 (35.6%) | 103(64.4%) | 0.313 |
| No favipiravir | | 2 (20%) | 8 (80%) |  |
| Remdesivir | | 3 (100%) | 0 (0%) | 0.040 |
| No remdesivir | | 56 (33.5%) | 111 (66.5%) |  |
| Hydroxychloroquine | | 6 (33.3%) | 12 (66.7%) | 0.897 |
| No hydroxychloroquine | | 53 (34.8%) | 99 (65.2%) |  |
| Famotidine | | 8 (32%) | 17 (68%) | 0.758 |
| No famotidine | | 51 (35.2%) | 94 (64.8%) |  |
| Colchicine | | 1 (33.3%) | 2 (66.7%) | 0.959 |
| No colchicine | | 58 (34.7%) | 109 (65.3%) |  |
| BL/BLI including empirical monotherapy | Present 18 (29.5%)  Absent 41 (37.6%) | Present 43 (70.5%)  Absent 68 (62.4%) | 0.286 |  |
| Carbapenem including empirical monotherapy | Present 6 (31.6%)  Absent 53 (35.1%) | Present 13 (68.4%)  Absent 98 (64.9%) | 0761 |  |
| Empirical combination therapy with quinolones | Present 8 (72.7%)  Absent 51 (32.1%) | Present 3 (27.3%)  Absent 108 (67.9%) | 0.006 |  |
| Antifungal including empirical therapy | Present 8 (34.7%)  Absent 51 (34.7%) | Present 15 (65.2%)  Absent 96 (65.3%) | 0.993 |  |
| Glycopeptide including therapy anytime during the COVID-19/FEN episode | Present 41 (55.4%)  Absent 18 (18.7%) | Present 33 (44.6%)  Absent 78 (81.3%) | <0.001 |  |
| BL/BLI including therapy anytime during the COVID-19/FEN episode | | Present 33 (35.5%)  Absent 26 (33.8%) | Present 60 (64.5%)  Absent 51 (66.2%) | 0.814 |
| Fluoroquinolone or macrolide including therapy anytime during the COVID-19/FEN episode | | Present 14 (45.2%)  Absent 45 (32.4%) | Present 17 (54.8%)  Absent 94 (67.6%) | 0.176 |
| Additional CDI | | Present 26 (42.6%)  Absent 33 (30.3%) | Present 35 (57.4%)  Absent 76 (69.7%) | 0.104 |
| Receiving two doses of COVID-19 vaccine before COVID-19 episode | | Present 3 (27.3%)  Absent 56 (35.2%) | Present 8 (72.7%)  Absent 103 (64.8%) | 0.592 |

| IVIG | Present 11 (50%)  Absent 48 (32.4%) | Present 11 (50%)  Absent 100 (67.6%) | 0.106 |
| --- | --- | --- | --- |
| Carbapenem including therapy anytime during the COVID-19/FEN episode | Present 48 (44.9%)  Absent 11 (17.5%) | Present 59 (55.1%)  Absent 52 (82.5%) | <0.001 |
| Supplemental oxygen | Present 57 (44.9%)  Absent 2 (4.6%) | Present 70 (55.1%)  Absent 41 (95.4%) | <0.001 |
| Mechanical  ventilation | Present 41 (93.2%)  Absent 18 (14.3%) | Present 3 (6.8%)  Absent 108(85.7%) | <0.001 |
| Septic shock during COVID-19/FEN | Present 24 (70.6%)  Absent 35 (25.7%) | Present 10 (29.4%)  Absent 101(74.3%) | <0.001 |
| High risk FEN MASCC (<21) | 36 (50.7%) | 35 (49.3%) | <0.001 |
| Low risk FEN MASCC (≥21) | 23 (23.2%) | 76 (76.8%) |  |
| One month mortality | Present 47 (61.8%)  Absent 12 (12.8%) | Present 29 (38.2%)  Absent 82 (87.2%) | <0.001 |
| Reinfection | Present 0 (0%)  Absent 59 (35.7%) | Present 5 (100%)  Absent 106 (64.3%) | 0.097 |
|  |  |  |  |
|  |  |  |  |

(PCR: polymerase chain reaction, BL/BLI: beta lactam beta lactamase inhibitor, FEN: febrile neutropenia, CDI: clinically documented infection, IVIG: intravenous immunoglobulin, ICU: intensive care unit, LDH: Lactic dehydrogenase, CRP: C reactive protein, COVID-19: Coronavirus disease 2019)

Table 4S: Univariate analysis for need for mechanical ventilation

| Parameter | | Needed mechanical ventilation  N: 44 | No need for mechanical ventilation  N: 126 | p |
| --- | --- | --- | --- | --- |
| Female | | 12 (20.7%) | 46 (79.3%) | 0.265 |
| Age | | 58.3 ± 15.4 | 59.6 ± 15.6 | 0.833 |
| Chronic renal failure | | Present 7 (100 %)  Absent 37 (22.7%) | Present 0 (0%)  Absent 126 (77.3%) | <0.001 |
| Non recovery from neutropenia | | 28 (52.8%) | 25 (47.2%) | <0.001 |
| Recovery from neutropenia | | 16 (13.7%) | 101 (86.3%) |  |
| The mean time for recovery from neutropenia | | 6.4 ± 6.7 | 5.3 ± 3.4 | 0.162 |
| COVID-19 convalescent plasma | | Present 7 (35%)  Absent 37 (24.7%) | Present 13 (65%)  Absent 113 (75.3%) | 0.321 |
| Bacterial coinfection | | Present 15 (28.3%)  Absent 29 (25.7%) | Present 42 (73.7%)  Absent 84 (74.3%) | 0.926 |
| Fungal coinfection (proven+probable+possible) n:40 | | Present 14 (35%)  Absent 30 (23.1%) | Present 26 (65%)  Absent 100 (76.9%) | 0.132 |
| Aspergillosis (Proven+ Probable+possible) n:27 | | Present 10 (37.1%)  Absent 34 (23.8%) | Present 17 (62.9%)  Absent 109 (76.3%) | 0.149 |
| Aspergillosis (Proven+ Probable) n:5 | | Present 2 (40%)  Absent 42 (25.5%) | Present 3 (60%)  Absent 123(74.5%) | 0.464 |
| Any proven fungal coinfection | | Present 5 (31.3%)  Absent 39 (25.3%) | Present 11 (68.7%)  Absent 115(74.7%) | 0.606 |
| Possible Fungal coinfection n:22 | | Present 8 (36.4%)  Absent 36 (24.3%) | Present 14 (63.6%)  Absent 112 (75.7%) | 0.228 |
| Age ≥60 | | Present 23 (23.9%)  Absent 21 (28.4%) | Present 73 (76.1%)  Absent 53 (71.6%) | 0.514 |
| Initial lymphocyte (/mm^3^) | | 1652 ± 7851 | 682 ± 902 | 0.173 |
| Initial lymphocyte <800/mm^3^ | | 38 (26.9%) | 103 (%73.1) | 0.491 |
| Initial lymphocyte ≥800/mm^3^ | | 6 (20.7%) | 23 (79.3%) |  |
| Ferritin <500 µg/L | | 9 (16.7%) | 45 (83.3%) | 0.052 |
| Ferritin ≥500 µg/L | | 29 (%31.2) | 64 (68.8%) |  |
| LDH≥250 U/L | | 28 (36.4%) | 49 (63.6%) | 0.022 |
| LDH<250 U/L | | 16 (20%) | 64 (80%) |  |
| LDH (U/L) | | 460 ± 429 | 318 ± 261 | 0.012 |
| Ferritin (µg/L) | | 2878 ± 3309 | 1790 ± 4765 | 0.195 |
| Ferritin <2000 µg/L | | 19 (18.3%) | 85 (81.7%) | 0.001 |
| Ferritin ≥2000 µg/L | | 19 (44.2%) | 24 (%55.8) |  |
| D-dimer (µg/L) | | 2644 ± 3684 | 1760 ± 3946 | 0.195 |
| D-dimer <1000 µg/L | | 14 (16.7%) | 70 (83.3%) | 0.004 |
| D-dimer ≥1000 µg/L | | 27 (36.9%) | 46 (63.1%) |  |
| D-dimer <2250 µg/L | | 28 (22.4%) | 97 (77.6%) | 0.036 |
| D-dimer ≥2250 µg/L | | 13 (40.6%) | 19 (59.4%) |  |
| Neutrophil count /mm^3^ | | 204 ± 169 | 282 ± 175 | 0.011 |
| Initial Neutrophil ≥250/mm^3^ | | 15 (16.7%) | 75 (83.3%) | 0.004 |
| Initial Neutrophil <250/mm^3^ | | 29 (36.3%) | 51 (63.7%) |  |
| CRP (mg/L) | | 141 ± 94 | 109 ± 113 | 0.093 |
| CRP ≥75 mg/L | | 34 (31.5%) | 74 (68.5%) | 0.027 |
| CRP <75 mg/L | | 10 (16.2%) | 52 (83.8%) |  |
| CRP ≥100 mg/L | | 32 (36.4%) | 56 (63.6%) | 0.001 |
| CRP <100 mg/L | | 12 (14.6%) | 70 (85.4%) |  |
| Underlying any solid tumors | | 11 (18.9%) | 47 (81.1%) | 0.039 |
| Underlying hematologic malignity | | 31 (33.3%) | 62 (66.7%) |  |
| No malignity | | 2 (10.5%) | 17 (89.5%) |  |
| PCR negativity during COVID-19 treatment | | Present 12 (17.9%)  Absent 6 (31.1%) | Present 55 (82.1%)  Absent 24 (68.9%) | 0.806 |
| Any steroid treatment in the overall cohort | | 32 (28.3%) | 81 (71.7%) | 0.307 |
| No steroid treatment in the overall cohort | | 12 (21.1%) | 45 (78.9%) |  |
| Any steroid treatment in O_2_ receiving cohort | | 32 (33.7%) | 63 (66.3%) | 0.694 |
| No steroid treatment in O_2_ receiving cohort | | 12 (37.5%) | 20 (62.5%) |  |
| Tocilizumab | | 6 (66.7%) | 3 (33.3%) | 0.010 |
| No tocilizumab | | 38 (23.6%) | 123 (76.4%) |  |
| Favipiravir | | Present 43 (26.9%)  Absent 1 (10%) | Present 117(73.1%)  Absent 9 (90%) | 0.237 |
| Remdesivir | | 3 (100%) | 0 (0%) | 0.016 |
| No remdesivir | | 41 (24.5%) | 126 (75.5%) |  |
| Hydroxychloroquine | | 5 (27.8%) | 13 (72.2%) | 0.846 |
| No hydroxychloroquine | | 39 (25.7%) | 113 (74.3%) |  |
| Famotidine | | 5 (20%) | 20 (80%) | 0.683 |
| No famotidine | | 39 (26.9%) | 106 (73.1%) |  |
| Colchicine | | 1 (33.3%) | 2 (66.7%) | 0.766 |
| No colchicine | | 43 (25.7%) | 124 (74.3%) |  |
| BL/BLI including empirical monotherapy | Present 11 (18.1%)  Absent 33 (30.3%) | Present 50 (81.9%)  Absent 76 (69.7%) | 0.080 |  |
| Carbapenem including empirical monotherapy | Present 5 (26.3%)  Absent 39 (25.8%) | Present 14 (73.7%)  Absent 112 (74.2%) | 0.002 |  |
| Empirical combination therapy with quinolones | Present 7 (63.6%)  Absent 37 (23.3%) | Present 4 (36.4%)  Absent 122 (76.7%) | 0.003 |  |
| Antifungal including empirical therapy | Present 7 (30.4%)  Absent 37 (25.2%) | Present 16 (69.6%)  Absent 110 (74.8%) | 0.591 |  |
| Glycopeptide including therapy anytime during the Fen+COVID-19 episode | Present 29 (39.2%)  Absent 15 (15.6%) | Present 45 (60.8%)  Absent 81 (84.4%) | <0.001 |  |
| BL/BLI including therapy anytime during the COVID-19/FEN episode | | Present 22 (23.7%)  Absent 22 (28.6%) | Present 71 (76.3%)  Absent 55 (71.4%) | 0.466 |
| Fluoroquinolone or macrolide including therapy anytime during the COVID-19/FEN episode | | Present 11 (35.5%)  Absent 33 (23.8%) | Present 20 (64.5%)  Absent 106 (76.2%) | 0.177 |
| Additional CDI | | Present 19 (31.2%)  Absent 25 (22.9%) | Present 42 (68.8%)  Absent 84 (%77.1) | 0.240 |
| Receiving two doses of COVID-19 vaccine before COVID-19 episode | | Present 3 (27.3%)  Absent 41(25.8%) | Present 8 (72.7%)  Absent 118 (74.2%) | 0.913 |

| IVIG | Present 10 (45.5%)  Absent 34 (22.9%) | Present 12 (54.5%)  Absent 114(77.1%) | 0.024 |
| --- | --- | --- | --- |
| Carbapenem including therapy anytime during the COVID-19/FEN episode | Present 27 (25.2%)  Absent 17 (%26.9) | Present 80 (74.8%)  Absent 46 (73.1%) | 0.801 |
| Supplemental oxygen | Present 44 (34.6%)  Absent 0 (0%) | Present 83 (65.4%)  Absent 43 (100%) | <0.001 |
| Septic shock during COVID-19/FEN | Present 22 (64.7%)  Absent 22 (16.2%) | Present 12 (35.3%)  Absent 114 (83.8%) | <0.001 |
| Need for ICU during COVID-19/FEN | Present 41 (69.5%)  Absent 3 (2.7%) | Present 18 (30.5%)  Absent 108(97.3%) | <0.001 |
| High risk FEN MASCC (<21) | 30 (42.3%) | 41 (52.7%) | <0.001 |
| Low risk FEN MASCC (≥21) | 14 (14.2%) | 85 (85.8%) |  |
| One month mortality | Present 42 (55.3%)  Absent 2 (2.2%) | Present 34 (44.7%)  Absent 92 (97.8%) | <0.001 |
| Reinfection | Present 0 (0%)  Absent 44 (26.7%) | Present 5 (100%)  Absent 121(73.3%) | 0.328 |
|  |  |  |  |
|  |  |  |  |

(PCR: polymerase chain reaction, BL/BLI: beta lactam beta lactamase inhibitor, FEN: febrile neutropenia, CDI: clinically documented infection, IVIG: intravenous immunoglobulin, ICU: intensive care unit, LDH: Lactic dehydrogenase, CRP: C reactive protein, COVID-19: Coronavirus disease 2019, MASCC: Multinational Association of Supportive Care in Cancer)

Table 5S: Univariate analysis for one month mortality: non-significant variables

| Parameter | | Day 30 survival  N: 94 | Day 30 mortality  N: 76 | p |
| --- | --- | --- | --- | --- |
| Female | | 36 (62.1%) | 22 (37.9%) | 0.201 |
| Age | | 57.9 ± 15.5 | 61± 15.4 | 0.1953 |
| The mean time for recovery from neutropenia | | 5.6 ± 4.3 | 5.4± 3.7 | 0.200 |
| Favipiravir | | Present 89 (55.7%)  Absent 5 (50%) | Present 71 (44.3%)  Absent 5 (50%) | 0.728 |
| COVID-19 convalescent plasma | | Present 9 (45%)  Absent 85 (56.7%) | Present 11 (55%)  Absent 65 (43.3%) | 0.324 |
| Bacterial coinfection | | Present 29 (50.8%)  Absent 65 (57.6%) | Present 28 (49.2%)  Absent 48 (42.4%) | 0.410 |
| Fungal coinfection (proven+probable+possible) n:40 | | Present 24 (60%)  Absent 70 (53.8%) | Present 16 (40%)  Absent 60 (46.2%) | 0.493 |
| Aspergillosis (Proven+ Probable+possible) n:27 | | Present 16 (75%)  Absent 78 (54.5%) | Present 11 (25%)  Absent 65 (45.5%) | 0.651 |
| Aspergillosis (Proven+ Probable) | | Present 4 (80%)  Absent 90 (54.5%) | Present 1 (20%)  Absent 75 (45.5%) | 0.259 |
| Any proven fungal coinfection | | Present 10 (62.5%)  Absent 84 (54.5%) | Present 6 (37.5%)  Absent 70 (45.5%) | 0.542 |
| Possible Fungal coinfection | | Present 12 (54.5%)  Absent 82 (55.4%) | Present 10 (45.5%)  Absent 66 (44.6%) | 0.939 |
| Age ≥60 | | Present 50 (52.1%)  Absent 44 (59.4%) | Present 46 (47.9%)  Absent 30 (40.6%) | 0.337 |
| Any underlying disease other than any malignity | | Present 53 (50.5%)  Absent 41 (63.1%) | Present 52 (49.5%)  Absent 24 (36.9%) | 0.108 |
| Hypertension | | Present 26 (52%)  Absent 68 (56.7%) | Present 24 (48%)  Absent 52 (43.3%) | 0.577 |
| COPD | | Present 5 (38.5%)  Absent 89 (56.7%) | Present 8 (61.5%)  Absent 68 (43.3%) | 0.204 |
| Diabetes mellitus (DM) | | Present 22 (62.9%)  Absent 72 (53.4%) | Present 13 (37.1%)  Absent 63 (46.6%) | 0.312 |
| Dyspnea at the time of COVID-19 diagnosis | | Present 25 (56.9%)  Absent 69 (54.7%) | Present 19 (43.1%)  Absent 57 (45.3%) | 0.813 |
| Cough at the time of COVID-19+FEN diagnosis | | Present 47 (49.5%)  Absent 47 (62.7%) | Present 48 (50.5%)  Absent 28 (37.3%) | 0.085 |
| Diarrhea at the time of COVID-19+FEN diagnosis | | Present 16 (57.2%)  Absent 78 (54.9%) | Present 12 (42.8%)  Absent 64 (45.1%) | 0.829 |
| Chest pain at the time of COVID-19+FEN diagnosis | | Present 16 (53.3%)  Absent 78 (55.7%) | Present 14 (46.7%)  Absent 62 (44.3%) | 0.811 |
| Headache at the time of COVID-19+FEN diagnosis | | Present 26 (65%)  Absent 68 (52.3%) | Present 14 (35%)  Absent 62 (47.7%) | 0.157 |
| Asymptomatic at the time of COVID-19+FEN diagnosis | | Present 12 (75%)  Absent 82 (53.2%) | Present 4 (25%)  Absent 72 (46.8%) | 0.095 |
| Initial leukocyte  (mean value at the start of treatment) | | 1232 ± 2219 | 2532 ± 9431 | 0.1975 |
| Severe neutropenia  (<100/mm^3^) | | Present 22 (46.8%)  Absent 72 (58.5%) | Present 25 (53.2%)  Absent 51 (41.5%) | 0.168 |
| Initial lymphocyte (/mm^3^) | | 652 ± 962 | 1281± 5978 | 0.3164 |
| Initial lymphocyte <800/mm^3^ | | 80 (56.7%) | 61 (43.3%) | 0.403 |
| Initial lymphocyte ≥800/mm^3^ | | 14 (48.3%) | 15 (51.7%) |  |
| Initial Neutrophil ≥250/mm^3^ | | 56 (62.23%) | 34 (37.3%) | 0.053 |
| Initial Neutrophil <250/mm^3^ | | 38 (47.8%) | 42 (52.2%) |  |
| Initial hemoglobin | | 7.3 ± 6.05 | 6.9 ± 3.4 | 0.6077 |
| Fibrinogen | | 341 ± 212 | 376 ± 241 | 0.3156 |
| Ferritin <500 µg/L | | 31 (57.4%) | 23 (42.6%) | 0.860 |
| Ferritin ≥500 µg/L | | 52 (55.9%) | 41 (44.1%) |  |
| LDH ≥250 U/L | | 40 (51.9%) | 37 (48.1%) | 0.484 |
| LDH<250 U/L | | 46 (57.5%) | 34 (42.5%) |  |
| Ferritin <2000 µg/L | | 68 (59.6%) | 46 (40.4%) | 0.147 |
| Ferritin ≥2000 µg/L | | 15 (45.4%) | 18 (45.5%) |  |
| D-dimer <2250 µg/L | | 75 (60%) | 50 (40%) | 0.097 |
| D-dimer ≥2250 µg/L | | 14 (43.7%) | 18 (56.2%) |  |
| Day 0 CT any involvement | | 74 (53.7%) | 64 (46.3%) | 0.093 |
| Day 0 CT no involvement | | 16 (72.7%) | 6 (27.3%) |  |
| ARDS in Day 0 CT | | Present 17 (44.7%)  Absent 77 (58.3%) | Present 21 (55.3%)  Absent 55 (41.7%) | 0.137 |
| Underlying any solid tumours | | Present 34 (58.6%)  Absent 60 (53.6%) | Present 24 (41.4%)  Absent 52 (46.4%) | 0.530 |
| Underlying hematologic malignity | | Present 50 (53.7%)  Absent 44 (57.1%) | Present 43 (46.3%)  Absent 33 (42.9%) | 0.659 |
| Underlying any solid tumors | | 34 (58.6%) | 24 (41.4%) | 0.817 |
| Underlying hematologic malignity | | 50 (53.7%) | 43 (46.3%) |  |
| No malignity | | 10 (52.6%) | 9 (47.4%) |  |
| Any bone marrow transplantation | | Present 4 (40%)  Absent 90 (56.3%) | Present 6 (60%)  Absent 70 (43.7%) | 0.316 |
| Allogenic bone marrow transplantation | | Present 3 (37.5%)  Absent 91 (56.1%) | Present 5 (62.5%)  Absent 71 (43.9%) | 0.299 |
| Autologous bone marrow transplantation | | Present 1 (50%)  Absent 93 (55.7%) | Present 1 (50%)  Absent 75 (44.6%) | 0.879 |
| Mean days after the last chemotherapy | | 31 ± 63.5 | 23.6 ± 53.6 | 0.4196 |
| PCR negativity during COVID-19 treatment | | Present 43(64.2%)  Absent 14 (46.7%) | Present 24 (35.8%)  Absent 16 (53.3%) | 0.105 |
| Any steroid treatment in the overall cohort | | 58 (51.3%) | 55 (48.7%) | 0.143 |
| No steroid treatmen t in the overall cohortt | | 36 (63.2%) | 21 (36.8%) |  |
| Pulse steroid in O_2_ receiving cohort | | 30 (49.2%) | 31 (50.8%) | 0.940 |
| No steroid treatment in O_2_ receiving cohort | | 16 (50%) | 16 (50%) |  |
| Pulse steroid in O_2_ receiving cohort | | 30 (49.2%) | 31 (50.8%) | 0.453 |
| Other steroid in O_2_ receiving cohort | | 14 (41.1%) | 20 (58.9%) |  |
| Any steroid treatment in O_2_ receiving cohort | | 44 (46.3%) | 51 (53.7%) | 0.718 |
| No steroid treatment in O_2_ receiving cohort | | 16 (50%) | 16 (50%) |  |
| 6 mg dexamethasone equivalent 30 mg methyl prednisolone in O_2_ receiving cohort | | 8 (53.3%) | 7 (46.7%) | 0.831 |
| No steroid treatment in O_2_ receiving cohort | | 16 (50%) | 16 (50%) |  |
| Pulse+other steroid in O_2_ receiving cohort | | 6 (26.1%) | 17 (73.9%) | 0.074 |
| No steroid treatment in O_2_ receiving cohort | | 16 (50%) | 16 (50%) |  |
| Tocilizumab | | 3 (33.3%) | 6 (66.7%) | 0.173 |
| No tocilizumab | | 91 (56.5%) | 70 (43.5%) |  |
| No favipiravir | | 9 (45%) | 11 (55%) | 0.605 |
| Favipiravir up to 5 days | | 60 (57.2%) | 45 (42.8%) |  |
| Favipiravir 10 or more days | | 25 (55.6%) | 20 (45.4%) |  |
| No favipiravir | | 9 (45%) | 11 (55%) | 0.316 |
| Favipiravir up to 5 days | | 60 (57.2%) | 45 (42.8%) |  |
| No favipiravir | | 9 (45%) | 11 (%55) | 0.431 |
| Favipiravir 10 or more days | | 25 (55.6%) | 20 (45.4%) |  |
| Favipiravir up to 5 days | | 60 (57.2%) | 45 (42.8%) | 0.857 |
| Favipiravir 10 or more days | | 25 (55.6%) | 20 (45.4%) |  |
| Remdesivir | | 0 (0%) | 3 (100%) | 0.0519 |
| No remdesivir | | 94 (56.2%) | 73 (43.8%) |  |
| Hydroxychloroquine | | 9 (%50) | 9 (%50) | 0.632 |
| No hydroxychloroquine | | 85 (55.9%) | 67 (44.1%) |  |
| Famotidine | | 11 (44%) | 14 (66%) | 0.218 |
| No famotidine | | 83 (57.2%) | 62 (42.8%) |  |
| Colchicine | | 2 (66.7%) | 1 (33.3%) | 0.689 |
| No colchicine | | 92 (55.1%) | 75 (44.9%) |  |
| BL/BLI including empirical monotherapy | Present 35 (57.4%)  Absent 59 (54.1%) | Present 26 (42.6%)  Absent 50 (45.9%) | 0.682 |  |
| Carbapenem including empirical monotherapy | Present 10 (52.6%)  Absent 84 (55.6%) | Present 9 (47.4%)  Absent 67 (44.4%) | 0.804 |  |
| Empirical combination therapy with quinolones | Present 7 (63.6%)  Absent 87 (54.7%) | Present 4 (36.4%)  Absent 72 (45.3%) | 0.565 |  |
| Antifungal including empirical therapy | Present 12 (52.2%)  Absent 82 | Present 11 (47.8%)  Absent 65 | 0.746 |  |
| Glycopeptide including therapy anytime during the COVID-19/FEN episode | Present 35 (47.3%)  Absent 59 (61.5%) | Present 39 (52.7%)  Absent 37 (38.5%) | 0.065 |  |
| BL/BLI including therapy anytime during the COVID-19/FEN episode | | Present 50 (53.8%)  Absent 44 (57.1%) | Present 43 (46.2%)  Absent 33 (42.9%) | 0.659 |
| Fluoroquinolone or macrolide including therapy anytime during the COVID-19/FEN episode | | Present 17 (54.9%)  Absent 77 (55.4%) | Present 14 (45.1%)  Absent 62 (44.6%) | 0.955 |
| Additional any bacterial infection | | Present 29 (50.8%)  Absent 65 (57.5%) | Present 28 (49.2%)  Absent 48 (42.5%) | 0.410 |
| Additional any MDI including possible and probable aspergillosis | | Present 53 (54.6%)  Absent 41 (56.2%) | Present 44 (45.4%)  Absent 32 (43.8%) | 0.843 |
| Additional MD bacteremia | | Present 19 (55.9%)  Absent 75 (55.1%) | Present 15 (44.1%)  Absent 61 (44.9%) | 0.938 |
| Additional MD UTI | | Present 14 (60.9%)  Absent 80 (54.4%) | Present 9 (39.1%)  Absent 67 (45.6%) | 0.563 |
| Additional CDI | | Present 33 (54.1%)  Absent 61 (55.9%) | Present 28 (45.9%)  Absent 48 (44.1%) | 0.14 |
| Additional MD pneumonia including probable Aspergillosis | | Present 11 (64.7%)  Absent 83 (54.2%) | Present 6 (35.3%)  Absent 70 (45.8%) | 0.410 |
| No additional MDI or CDI (FUO except COVID-19)) | | Present 25 (50%)  Absent 69 (57.5%) | Present 25 (50%)  Absent 51 (42.5%) | 0.370 |
| Receiving two doses of COVID-19 vaccine before COVID-19 episode | | Present 7 (61.5%)  Absent 87 (54.7%) | Present 4 (38.5%)  Absent 72 (45.3%) | 0.565 |

(COPD: chronic obstructive pulmonary disease, DM: diabetes mellitus, FEN: febrile neutropeniae, CT: Computed tomography, ARDS: acute respiratory distress syndrome, PCR: polymerase chain reaction, BL/BLI: beta lactam beta lactamase inhibitor, MDI: microbiologically diagnosed infection, MD: microbiologically defined, UTI: urinary tract infection, CDI: clinically documented infection, FUO: fever of unknown origin COVID-19: Coronavirus disease 2019, LDH: Lactic dehydrogenase)

Table 6S: Logistic regression analysis results for PCR negativity during the COVID-19 and febrile neutropenia episode

| **Covariate** | **Odds ratio** | **95% CI** | **p** |
| --- | --- | --- | --- |
| **Receiving no IVIG** | 3.881 | 0.779-19.320 | 0.098 |
| **Receiving up to 5 days of favipiravir** | 5.166 | 1.639-16.280 | 0.005 |

(PCR: polymerase chain reaction, IVIG: intravenous immunoglobulin)

Table 7S: Logistic regression analysis outcomes results for the need for ICU during the COVID-19/FEN episode

| **Covariate** | **Odds ratio** | **95% CI** | **p** |
| --- | --- | --- | --- |
| **CRP**≥**100 mg/L** | 1.736 | 0.598-5.039 | 0.310 |
| **Supplemental oxygen** | 4.005 | 0.723-22.179 | 0.112 |
| **Mechanical ventilation** | 62.042 | 9.528-404.011 | <0.001 |
| **Initial neutrophil count below 250/mm^3^** | 1.511 | 0.481-4.744 | 0.480 |
| **High risk FEN (MASCC <21)** | 1.568 | 0.498-4.942 | 0.442 |
| **Septic shock during COVID-19/FEN episode** | 2.138 | 0.496-9.220 | 0.308 |
| **Lack of recovery from neutropenia** | 0.641 | 0.173-2.376 | 0.506 |
| **Any steroid** | 3.135 | 0.827-11.884 | 0.093 |
| **Glycopeptide including therapy anytime during the COVID-19/FEN episode** | 6.566 | 2.137-20.172 | 0.001 |
| **No PCR negativity during COVID-19 treatment** | 1.511 | 0.329-6.927 | 0.674 |
| **Receiving tocilizumab** | 0.800 | 0.029-21.773 | 0.895 |
| **Chronic renal failure** | 0.735 | 0.036-15.005 | 0.841 |

(PCR: polymerase chain reaction, CRP: C reactive protein, COVID-19: Coronavirus disease 2019

FEN: febrile neutropenia, MASCC: Multinational Association of Supportive Care in Cancer

PCR: polymerase chain reaction)

Table 8S: Logistic regression analysis outcomes results for the need for mechanical ventilation during the COVID-19/FEN episode

| **Covariate** | **Odds ratio** | **95% CI** | **p** |
| --- | --- | --- | --- |
| **Lack of recovery from neutropenia** | 17.869 | 3.592-88.907 | <0.001 |
| **Having an underlying hematological malignancy** | 1.575 | 0.288-8.624 | 0.600 |
| **Receiving tocilizumab** | 32.227 | 1.469-707.053 | 0.028 |
| **Glycopeptide including therapy anytime during the COVID-19/FEN episode** | 0.585 | 0.135-2.535 | 0.474 |
| **Receiving IVIG** | 4.412 | 0.514-37.834 | 0.176 |
| **Septic shock during COVID-19/FEN episode** | 15.496 | 3.164-75.897 | 0.001 |
| **Need for ICU during COVID-19/FEN episode** | 91.818 | 15.360-548.873 | <0.001 |
| **High risk FEN (MASCC <21)** | 1.551 | 0.359-6.703 | 0.557 |
| **Initial Neutrophil<250/mm^3^** | 1.658 | 0.326-8.444 | 0.542 |
| **CRP ≥100 mg/L** | 1.559 | 0.374-6.498 | 0.542 |

(ICU: intensive care unit, CRP: C reactive protein, COVID-19: Coronavirus disease 2019, FEN: febrile neutropenia, MASCC: Multinational Association of Supportive Care in Cancer, IVIG: intravenous globulin)

Table 9s: Logistic regression analysis outcomes results for one month mortality during the COVID-19 and febrile neutropenia episode

| **Covariate** | **Odds ratio** | **95% CI** | **p** |
| --- | --- | --- | --- |
| **Septic shock during COVID-19/FEN episode** | 5.125 | 1.435-18.306 | 0.012 |
| **Mechanical ventilation** | 22.904 | 4.211-124.573 | <0.001 |
| **High risk FEN MASCC (<21)** | 1.731 | 0.741-4.044 | 0.205 |
| **Need for ICU during COVID-19/FEN** | 1.938 | 0.661-5.679 | 0.228 |
| **Receiving IVIG** | 2.452 | 0.682-8.813 | 0.169 |
| **Supplemental oxygen** | 0.973 | 0.364-2.601 | 0.956 |
| **Carbapenem including therapy anytime during the COVID-19/FEN episode** | 1.052 | 0.447-2.474 | 0.907 |
| **CRP**≥**75 mg/L** | 1.687 | 0.680-4.181 | 0.259 |

(ICU: intensive care unit, FEN: febrile neutropeniae, IVIG: intravenous immunoglobulin, CRP: C reactive protein, MASCC: Multinational Association of Supportive Care in Cancer)
